# The Use of Pepsine in “Alimentation in Disease”

**Published:** 1868-07

**Authors:** Jas. S. Hawley


BUFFALO

Medical and Surgical Journal.

VOL. VII. JULY, 1868. No. 12.

### Original Communications.

ART. I. *The Use of Pepsine in "Alimentation in Disease."* By JAS. S. HAWLEY, M. D.

One of the most marked changes which has come over the practice of medicine during the last quarter of a century is the diminished confidence in the power of drugs to antagonize or remove disease. The advanced physician of the present day prescribes few and simple medicines; his efforts are protective rather than curative; he does not so much attempt to destroy the disease as to protect his patient against its ravages; his efforts are conservative more than aggressive.

Perhaps, in no one particular is this change more perceptible than in the prominence given to alimentation in disease. The importance of this subject is most clearly and ably set forth in a lecture delivered by Prof. Austin Flint, Sr., and published in the New York *Medical Journal.*

He says "starvation may be produced in persons affected with different diseases, as well as in healthy persons. There is nothing in disease to prevent starvation or its immediate effects. Starvation is sure to occur in cases of disease in a degree proportionate to the lack of material for nutrition in the blood; in other words, in proportion as the requisite amount of aliment is either not injected or not assimilated.

"The immediate effects and the attendant phenomena are the same where starvation occurs in connection with disease, as when it is produced in persons previously in health. Impoverishment of the blood, emaciation, febrile movement followed by a reduction of the animal temperature, feebleness of the circulation, vigilance, perversion of the moral sentiment, delirium, diarrhœa, and foetor of the breath may be attributable in cases of disease to starvation. In connection with all diseases, more or less of the morbid phenomena present arise from starvation, and these phenomena are prominent and grave in proportion to the degree in which either alimentation or assimilation is defective.

Chossat has enunciated truths in language which enforces their importance when he says, "Starvation is a cause of death, marching silently in front of every disease in which alimentation falls below the natural standard. It reaches its natural termination sometimes sooner and sometimes later than the disease which it covertly accompanies; and it may supercede the disease, of which, at first, it was merely an incidental element." Starvation is often the immediate cause of death, when diseases destroy life by slow asthenia or exhaustion.

If a fatal termination be not due to a direct interference with the action of the heart, or with respiration, it is correct to say that the patients die because they are starved to death. Certain it is that diseases which do not compromise directly the function of either the heart or lungs, *cannot kill so long as the nutrition of the body is maintained at a point compatible with life.* Starvation, associated with disease, may be inevitable; that is, the disease may involve an insuperable obstacle either to the ingestion of aliment or its assimulation. Then it is that, in the language of Chossat, inanition may reach its termination sooner than the disease. On the other hand, and here is a fact full of practical import; starvation may not be a necessary effect of the existing disease, but may be due to insufficient alimentation. In such cases *inanition may prove a cause of death when the disease need not have destroyed life; the patient, indeed, may die of starvation, notwithstanding the progress of the disease per se, be favorable.* Then, in the language of Chossat, "inanition reaches its natural termination later than the disease which it covertly accompanies, and it may supercede the disease of which at first it was merely an incidental element."

The importance of this object in the treatment of individual cases of disease, is to be estimated by the amount of impending danger from starvation as an incidental element. If to die by slow asthenia be often virtually to starve to death, then no matter what the disease may be, *it is an object of fundamental importance to promote, as far as practicable, the assimilation of blood.*

In acute disease the failure of the vital powers is forestalled in proportion as nutritive supplies are assimilated. This is simply saying that *the assimulation of nourishment is indispensable for the preservation of the powers of life.*

No matter what may be the seat or nature of the chronic affection, a diet fully up to the capacity of the organism for nutrition promotes recovery, if recovery be possible, and if recovery be not possible, by increasing the ability of the system to endure the affection, contributes to prolong life. *The limitations to alimentation, therefore, relate wholly to the physiological processes which are preliminary to nutrition, namely: digestion and the other processes by which aliment is converted into food.*

I have made these lengthy quotations because they graphically set forth the importance of alimentation in disease, in much stronger language than I could command, and because the high source from which they proceed give them great weight.

Now, if nutrition holds the rank in the treatment of disease which is above set forth, then any agent or article of the materia medica which will enable the practitioner to increase the power of digestion and assimilation in the patient, must be acknowledged of primary importance. The difficulty to be encountered, is not generally in the ingestion of food. The patient retains the power of deglutition long after he has lost or suffered the impairment of his digestion and assimulation. And here we come to the object of this communication, to carry the point urged by Prof. Flint, one step further, and resort to artificial digestion. The skill and perfection with which the gastric fluid of animals is now extracted and preserved for medicinal use, puts it in our power to assist the sufferer at any and every stage of his struggle with disease. Pepsine may be administered with his food and new power and efficiency imparted to his failing functions.

To whatever degree starvation may be a cause of death in disease, to whatever extent disease may overwhelm the powers of life in consequence of insufficient nutrition to that degree and to that extent is artificial digestion important. We may, and do, "feed fevers," we do attempt to mitigate the emaciation of the consumptive by the administration of cod liver oil, but how painfully do we often witness their failure in consequence of the impairment of the digestive powers of our patients. Here, pepsine freely administered, is our only resource. Nor is this merely a temporary expedient. The stomach participates in the nutrition which it has been enabled to accomplish. Its own natural powers are gradually restored, and at length it performs its office unassisted. The use of pepsine also frequently prepares the way to the successful administration of other medicines, as is exhibited by the following quotation from Dr. Chambers' Renewal of Life. Speaking of the administration of pepsin in tubercular consumption, he says: "You will generally find that the repugnance of the patient to meat has been overcome, and that a small quantity of it at a time can be relished and digested; the morbid fetor of the stools diminishes, and flatulence and distress arising during their passage through the bowels ceases. A renewed strength and a renewed power of assimilation commences, the sleep becomes more natural with the diminution of night sweats and hectic; while at the same time the pulmonary symptoms of cough, dyspnoœa, etc., relax, and a step at any rate is taken in the right direction towards the cure of the disease. *It is remarkable too, what a slight improvement in the digestive powers will often enable the patient to take iron and cod liver oil.* These are, you know, the main stays in the treatment of tubercular consumption, and *any expedient, however temporary, which will pave the way for their administration is a great boon.*"

We cannot even know the natural history of disease until we have eliminated those elements and phenomena which appertain to starvation or deficiency in assimilation. In many instances this feature in the progress and development of disease can only be removed by the successful accomplishment of artificial digestion. In thousands of instances, doubtless, physicians imagine that they are liberally supporting their patients, when in fact they are filling their stomachs with substances which, from lack of ability to digest them, become foreign bodies, undergoing decomposition, engendering offensive and injurious gases, and inducing by their presence irritation of the entire alimentary canal.

It is not necessary further to push this argument. The object of this communication was confined to one point, the mere successful accomplishment of "alimentation in disease by artificial digestion." The only question remaining to be answered to complete the argument is this: Can artificial digestion be successfully accomplished by the administration of pepsine? Undoubtedly it can. This is now a matter of recorded experience, sufficiently extensive to remove all doubt. Doubtless many have met with disappointment. Many articles of pepsine are inert and unworthy of confidence, but with an active preparation success is certain.

